# Supplementary material for: The genome sequence of the Acorn Weevil, Curculio glandium (T.Marsham, 1802) (Coleoptera: Curculionidae)
Source: Wellcome Open Res. 2026 Mar 18;11:178. [Version 1] doi: 10.12688/wellcomeopenres.26157.1 (PMC13096787; doi:10.12688/wellcomeopenres.26157.1)
Supplement: Peer review history

# Reviewer report: Reviewer response for version 1

**DOI:** 10.21956/wellcomeopenres.28805.r151236

**Published:** 2026-04-20

**Authors:** Maria Antonia Madrid Restrepo

**Affiliations:** 1 KU Leuven, Leuven, Belgium

This data note presents a chromosome-level genome assembly for the acorn weevil *Curculio glandium*, generated within the Darwin Tree of Life project. The authors assembled a 1.12 Gb genome from a female specimen collected in Wytham Woods, with 97.77% of the sequence assigned to 13 chromosomal pseudomolecules, including the X chromosome, and they also recovered the mitochondrial genome. The study combines PacBio HiFi, Hi-C, and RNA-seq data, and reports strong assembly statistics, including a scaffold N50 of 99.31 Mb, BUSCO completeness of 98.5%, and a combined assembly QV of 59.7. Overall, this is a useful and technically solid genomic resource that will be valuable for comparative genomics and future work on Curculionidae. The rationale for creating the dataset is clearly described. The protocols are appropriate and the work appears technically sound. The methods and materials are, in general, described in sufficient detail to allow replication. The manuscript provides specimen identifiers, sequencing accessions, software names and versions, and the main analytical steps used in processing and assembly. Public deposition of raw reads and assembly files also supports reuse. The datasets are clearly presented and accessible. 

 First, the authors should correct the obvious affiliation error listing “Welshpool, Wales, USA”; this should be changed to “Welshpool, Wales, UK.” This is minor and editorial, but it should be fixed in the revised version. Second, the introduction would benefit from a brief comparison with existing genomic resources for related weevil species, especially any available *Curculio* or closely related Curculionidae assemblies.  Third, the statement that a “haplotypic inversion was observed” on chromosome 3 would benefit from a little more explanation. At present, the manuscript notes the inversion interval but does not explain how confidently this represents a biological haplotypic inversion rather than an assembly or phasing feature. Points that should be addressed to make the article scientifically sound: in my view, none of the issues rise to the level of a major scientific flaw, and the article is already scientifically sound as a genome data note. The only point that clearly must be corrected is the affiliation error.

Are sufficient details of methods and materials provided to allow replication by others?

Yes

Is the rationale for creating the dataset(s) clearly described?

Yes

Are the datasets clearly presented in a useable and accessible format?

Yes

Are the protocols appropriate and is the work technically sound?

Yes

Reviewer Expertise:

Eco-evolutionary genomics, computational biology, population genetics, bioinformatics

I confirm that I have read this submission and believe that I have an appropriate level of expertise to confirm that it is of an acceptable scientific standard.

# Reviewer report: Reviewer response for version 1

**DOI:** 10.21956/wellcomeopenres.28805.r151239

**Published:** 2026-04-03

**Authors:** Min Li

**Affiliations:** 1 Taiyuan Normal University, Jinzhong, China

 This manuscript is a high-quality data note reporting a chromosome-level genome assembly of the acorn weevil *Curculio glandium*. The work follows standard best practices for eukaryotic genome sequencing and assembly, produces a robust, well-validated genomic resource, and fully meets the publication criteria for Wellcome Open Research. I suggest only a few minor changes:

1. Affiliation 4: Welshpool, Wales, USA → Welshpool, Wales, UK.
2. The introduction lacks a brief summary of existing genomic resources for closely related Curculio species. To add 1–2 sentences comparison would strengthen the novelty of this chromosome-level assembly.

Are sufficient details of methods and materials provided to allow replication by others?

Yes

Is the rationale for creating the dataset(s) clearly described?

Yes

Are the datasets clearly presented in a useable and accessible format?

Yes

Are the protocols appropriate and is the work technically sound?

Yes

Reviewer Expertise:

NA

I confirm that I have read this submission and believe that I have an appropriate level of expertise to confirm that it is of an acceptable scientific standard.
